# Nothing New under the Sun—(Pompeian Instruments)

**Published:** 1830-01-01

**Authors:** 


### XLII.

### NOTHING NEW UNDER THE SUN.

In a late excursion to POMPEII, and examination of the various antiquities rescued from the oblivion of two thousand years beneath the ashes of Vesuvius, the Editor of this Journal was much interested by the numerous chirurgical instruments of our Pompeian forefathers collected in the Museum of Naples. His attention was particularly arrested by WEISS'S DILATOR, the original of which may there be seen, so precisely similar to that manufactured in the Strand, that, excepting the handles (one of which is in bronze and the other in ivory) it would be extremely difficult to distinguish the ancient from the modern INVENTION. Upon expressing his surprise at this remarkable coincidence, after a lapse of twenty centuries, the Curator of the Studii (the learned Abbé Jorio) observed that it was probably no coincidence but a *consequence*. He informed Dr. Johnson that, about ten or twelve years ago, a French gentleman took a memorandum of the instrument in question, and soon afterwards brought out at Paris a *modification* of the Pompeian Speculum or Dilator.* Now Mr. Weiss, while improving on the Parisian invention, did actually stumble upon the plan of the original instrument, so that, if the handles were of the same materials, it would be impossible to say which was the elder. It appears from this that of the two modern inventors, Mr. Weiss is the more original and ingenious. The Parisian disguised the model from which he worked, and made a clumsy

---

* The Abbé Jorio, the gentleman in question, has put the following note at the foot of page 116 of his account of Pompeii.

"Cet interessant instrument (speculum matricis) a été publié par une étranger, mais le dessin en ayant été *fait de memoire*, n'est pas du tout exact."—PLAN DE POMPEII.

The "*fait de memoire*" is, we fear, a too amiable construction of the *deviation* which the French mechanist made in the instrument. The want of candour, however, has been punished by the want of success—for had he constructed the instrument precisely as it now exists in the Museum of Naples, it would have been impossible to improve upon the plan.—J. J.

instrument—Weiss, in his endeavour to improve on the furtive copy, ascended, unconsciously, to the merits of the original!*

Among the Pompeian instruments there is a trocar exactly of the modern shape and size. The catheters are made of bronze, and very slightly curved, having an eye on one side, like our modern elastic catheters. There are some of these instruments without any curve whatever—shewing that the Ancients knew the practicability of introducing the straight staff.

The ancients seem to have been perfectly well acquainted with the *vapour-bath*. At Pompeii, Dr. J. examined one which is on a magnificent scale, and admirably adapted for the purpose of a public bath. From a very fine room, heated by braziers, an entrance leads to the CALIDARIUM, or vapour-bath, whose walls, floor, and ceiling, are double, and capable of being filled with vapour from two or three cauldrons, by means of leaden tubes. The vapour is admitted into the room itself from the hollow walls, &c. by small capillary apertures, while, at one end of the room issues forth, as from a fountain, a jet of boiling water, diffusing still more vapour through the apartment. Seats are ranged around for those who take the bath, and when finished, they retire into the room heated with warm air to dry and clothe themselves.

While observing the ingenuity of the Pompeians, it is impossible not to conclude that they were a most degenerate and depraved people. The figures portrayed in fresco on the walls even of the best houses, exhibit melancholy and disgusting proofs of the horrible depth of infamy, and even bestiality, into which they were sunk! It was high time that, like Sodom and Gomorrah, the cities of Herculaneum and Pompeii should be visited by fire and brimstone, to put a period to their iniquities, and draw the veil of oblivion over their obscenities! That veil, however, has been removed; and an awful catastrophe has preserved more unequivocal proofs and portraits of the private habits of the Italians than the pages of their best historians! The Pompeians have been doubly unfortunate. They were smothered in the ashes of Vesuvius, and they were destined to be exhumated, eighteen centuries afterwards, as specimens of the degeneracy of their times.

* Dr. Johnson called on Mr. Weiss a few days ago, and mentioned the circumstance of his dilator being a precise copy of that found in the ruins of Pompeii—at which the ingenious mechanic was not less astonished than gratified. That *he* did *not* benefit by the original is quite evident by the number of modifications which he manufactured (and which he still preserves, before he arrived at the present form.—J. J.

---

